# A Rich-Club Organization in Brain Ischemia Protein Interaction Network

**DOI:** 10.1038/srep13513

**Published:** 2015-08-27

**Authors:** Ali Alawieh, Zahraa Sabra, Mohammed Sabra, Stephen Tomlinson, Fadi A. Zaraket

**Affiliations:** 1 Department of Neurosciences, Medical University of South Carolina, Charleston, SC 29425; 2 Department of Electrical and Computer Engineering, American University of Beirut, Beirut, Lebanon; 3 Department of Microbiology and Immunology, Medical University of South Carolina, Charleston, SC 29425.

## Abstract

Ischemic stroke involves multiple pathophysiological mechanisms with complex interactions. Efforts to decipher those mechanisms and understand the evolution of cerebral injury is key for developing successful interventions. In an innovative approach, we use literature mining, natural language processing and systems biology tools to construct, annotate and curate a brain ischemia interactome. The curated interactome includes proteins that are deregulated after cerebral ischemia in human and experimental stroke. Network analysis of the interactome revealed a rich-club organization indicating the presence of a densely interconnected hub structure of prominent contributors to disease pathogenesis. Functional annotation of the interactome uncovered prominent pathways and highlighted the critical role of the complement and coagulation cascade in the initiation and amplification of injury starting by activation of the rich-club. We performed an in-silico screen for putative interventions that have pleiotropic effects on rich-club components and we identified estrogen as a prominent candidate. Our findings show that complex network analysis of disease related interactomes may lead to a better understanding of pathogenic mechanisms and provide cost-effective and mechanism-based discovery of candidate therapeutics.

Ischemic stroke still has the highest burden among all neurological diseases despite tremendous efforts devoted to prevention, management, treatment and rehabilitation of stroke patients^1^,^2^. Brain ischemia is characterized by reduction in blood flow to the brain resulting in unmet metabolic demands, tissue infarction and cell death. Ischemia is commonly followed by restoration of blood supply, i.e. reperfusion, either spontaneously or pharmacologically leading to activation of blood-derived pro-inflammatory components and secondary injury^3^. The short time in which events develop, as well as the multitude of consequent pathogenic mechanisms that arise after ischemia and reperfusion, make the treatment of this disease a challenge^4^,^5^. Preclinical and clinical studies have predicted that a single-action-single-target paradigms are not the optimal approach to treat stroke and that multi-action-multi-target paradigms will be required^6^. Such an approach requires the compilation of efforts in order to understand the evolution of different mechanisms after ischemic stroke and the relationship of various mechanisms to disease outcome and potential interventions. Thus, further progress in enhancing ischemic stroke management necessitates an understanding of the multiple interacting mechanisms that occur after stroke onset.

Network analysis tools were previously used to analyze biological networks including protein-protein interaction networks and neuronal connectivity networks^7^,^8^,^9^. For instance, topological analyses provided a more profound understanding of brain connectivity network through the discovery of a rich-club organization in the cat brain connectome^10^ that preceded the discovery of a similar rich-club in the human connectome^8^. This rich-club serves as a high capacity backbone system critical for physiological neuronal connectivity. Therefore, we hypothesize that the use of network analysis tools in the context of stroke protein interactome will provide a deeper understanding of the sequel of pathological events that happen after ischemia, and point out potential avenues for therapeutic interventions.

In this work, we describe a novel strategy using a semi-automatic annotation and text-mining approach coupled to systems biology and network analysis to analyze the complex protein interaction network that occurs after stroke. We curated and annotated a brain-ischemia interactome (BII) referring to set of interactions among proteins reported to exhibit changes in levels or regulation after human or experimental stroke. Network analysis uncovered a rich-club organization in the BII and provided insight into the predominating mechanisms in the early and subsequent phases of ischemic stroke. In addition, drug-protein interaction networks were used as an *in-silico* screening tool for putative therapeutic interventions that target the stroke rich-club.

## Results

### Curation and Annotation of First Brain Ischemia Interactome

A total of 82,181 articles were screened for including data on changes in the levels or regulation of gene products after brain ischemia using our semi-automatic annotation approach (Supplementary Figures 1 and 2). A total of 8,740 papers were selected through the initial screening and gene products reported in these studies are included in the Brain Ischemia Interactome (BII). Included gene products are those reported to have increased levels, decreased levels, or changes in localization or regulation (post-transcriptional or post-translational) after brain ischemia. Supplementary Table 1 summarizes proteins with highest frequency of occurrence in stroke literature. Tissue-plasminogen activator (t-PA) was the most frequently reported protein in the interactome, and its recombinant form is currently the only approved pharmaceutical intervention for acute stroke.

**Figure 1:**
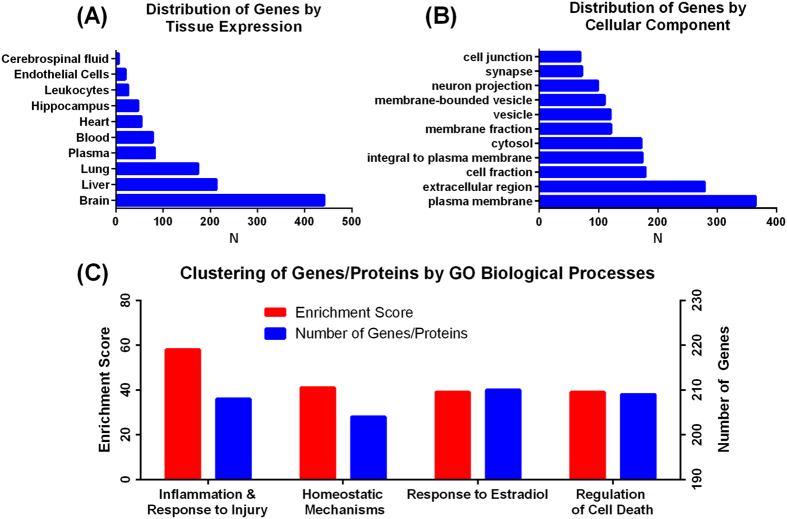
Functional annotation of brain ischemia interactome proteins. (**A**) Functional annotation of BII proteins by tissue expression reveals a predominant expression in brain tissue followed by liver tissue. This finding is anticipated given the fact that stroke is a disease of brain tissue that also involves systemic response mechanisms. Blue bars indicate the number of genes per annotation category enriched in BII with less than 1% FDR. (**B**) Functional annotation of BII proteins by cellular components reveals that the majority of the proteins are present in the extracellular space and plasma membrane compared to cytosol and cellular fractions indicating that the majority of pathophysiological events after stroke occur on and around the cell surface. Blue bars indicate the number of genes per annotation category enriched in BII with less than 1% FDR. (**C**) Clustering of enriched GO biological processes shows that inflammatory processes were the most enriched biological processes followed by homeostatic mechanisms, and then response to estradiol and regulation of cell death. Red bars show the enrichment score calculated through functional annotation clustering in DAVID. Blue bars show the number of genes for each functional annotation.

The BII was built using data on protein-protein interactions from STRING (Search Tool for the Retrieval of Interacting Genes/Proteins) database including all connections with a STRING combined score higher than 0.4 as previously described^11^,^12^. The resulting curated interactome consisted of 886 proteins connected by 17,425 binding interactions. Functional annotation and clustering of proteins in the BII were performed using DAVID (Database for Annotation, Visualization and Integrated Discovery) for enriched GO (Gene Ontology) biological processes, cellular components and tissue expressions^13^. Enrichment analysis was performed to identify processes, pathways and gene categories that are over-represented in the BII compared to the full human genome. As summarized in Fig. 1, BII proteins are predominantly expressed by brain tissue (**1A**), and are preferentially present at the plasma membrane and extra-cellular space **(1B)**. Clustering for GO enriched biological processes reveals that inflammatory responses are the most enriched processes (Fig. 1C).

**Figure 2:**
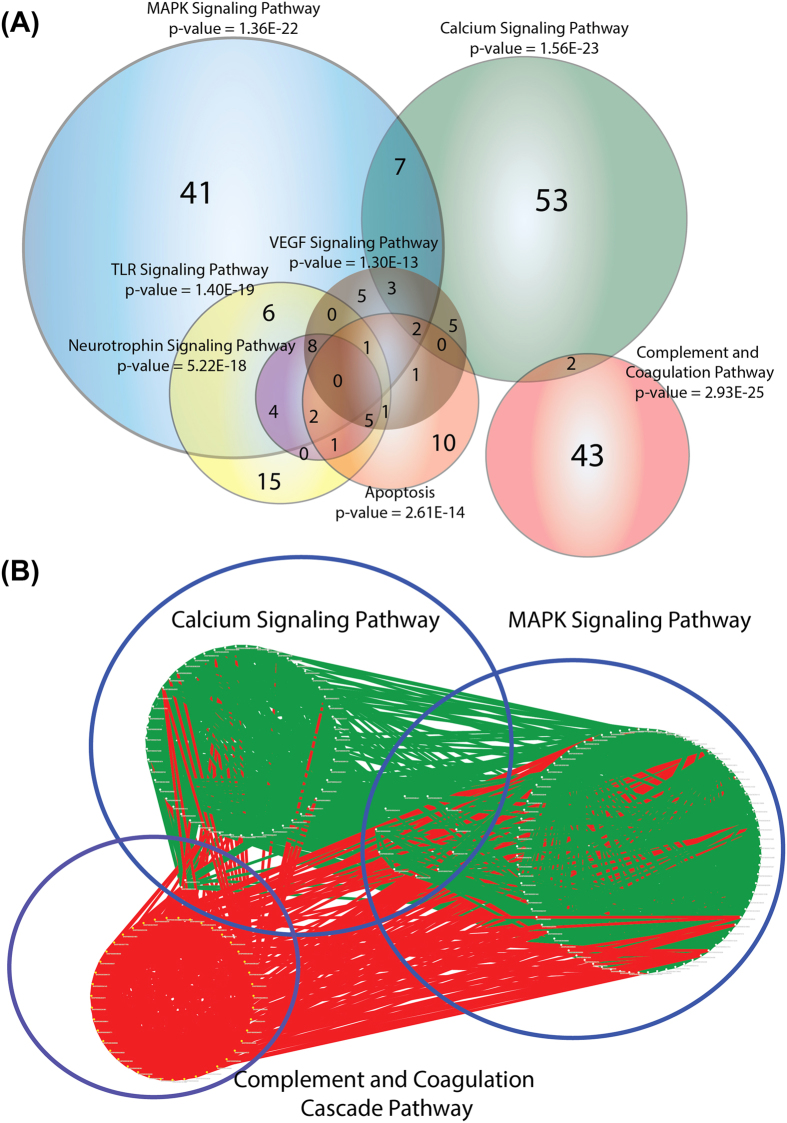
Venn Diagram of the distribution of BII proteins on different significantly enriched KEGG pathways. (**A**) Pathways with p-value less than 10^–12 are included. Complement and coagulation cascade (CCC) is the most enriched pathway and together with calcium signaling and MAPK signaling pathways form the three most significant pathways in our BII. Notably, Complement and Coagulation Pathway has little overlap in terms of components (4.4%) with other pathways compared to the latter two major pathways (22% and 48%). (**B**) Protein -protein interactions among the three most prominent pathways in the network. White dots indicate a node (protein) and edges indicate interactions. Red edges denote interactions that involve the CCC. Other edges are colored green. Despite the minimal intersection in terms of components between the CCC and other pathways, this cascade is still heavily interconnected with other prominent pathways in the network.

KEGG (Kyoto Encyclopedia of Genes and Genomes)^14^ pathway annotation revealed that complement and coagulation cascade (CCC) was the most enriched pathway followed by calcium signaling and mitogen-activated-kinase (MAPK) pathways. Notably, there was minimum overlap between components of the CCC pathway and other major enriched pathways in the network (Fig. 2A). Supplementary Figure 3 shows the identity of proteins in the BII that belong to the enriched pathways in Fig. 2.

To assess whether components of the CCC pathway were isolated within the network compared to components of other pathways, protein-protein interactions (PPI) between CCC pathway proteins and proteins of other prominent pathways discovered by KEGG annotation were analyzed. Results showed that components of the CCC pathway were heavily interconnected with proteins in other pathways (Fig. 2B), a finding that is specifically significant given the early role of this pathway in the recognition and response to ischemic and reperfusion injury^3^,^15^.

### Rich-Club Organization in Brain Ischemia Interactome

**Figure 3:**
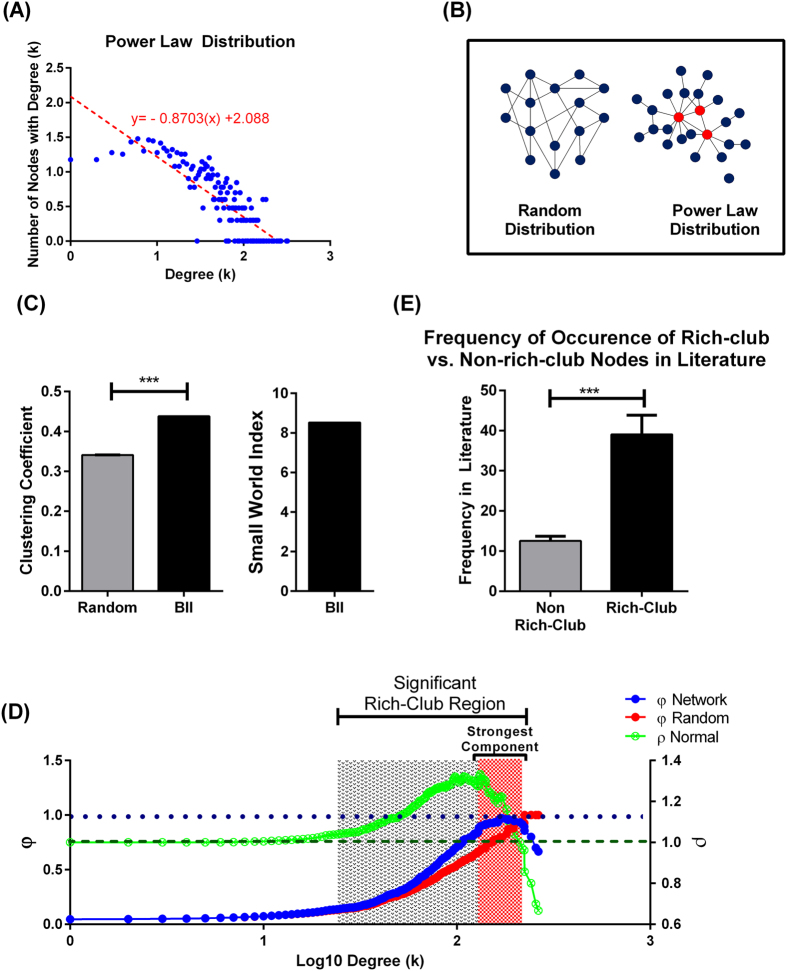
Properties of the BII Network. (**A**) Power-law distribution curve of the BII network shows a negative correlation between node frequency (vertical axis) and node-specific degree (horizontal axis). This indicates that there are low frequency of nodes with higher degree in the network (hubs) and high frequency of low degree nodes (non-hubs). (**B**) Example of a power-law network compared to random network. Circles denote nodes in the network, red circles denote hub nodes, and blue circles denote non-hub nodes. (**C**) Identification of small-world organization within the BII. Clustering coefficient of BII network was significantly higher than that of randomly generated comparable networks (n=100). The small-world coefficient was 8.5 indicating the presence of a small world organization. One-sample t-test; ***p-value < 0.0001. (**D**) Raw rich-club coefficient of our network (blue) and random network (red) plotted against the left vertical axis. Normalized rich-club coefficient for the network (green) plotted against the right vertical axis. The shaded region indicates the range of degrees over which a rich-club organization is present (degree 40–180; peak at degree 132). The region of strongest rich-club component is also highlighted in red. Horizontal dashed lines correspond to unity values of 1 for both φ and ρ. (**E**) Nodes constituting the rich-club were significantly more studied (higher frequency of occurrence) in the curated literature than nods outside the rich club. *Bars* = *mean* +/− SEM. ***p < *0.0001*.

Network analysis of the BII revealed that it exhibits a power-law degree distribution consistent with being a scale-free network, a property of most biological networks. Figure 3A shows that the frequency of nodes with certain degree (k) is inversely correlated with the degree (k) indicating that a few number of nodes have the majority of the interactions in the network and are thus hub nodes (illustrated in Fig. 3B). Further analysis of the network clustering coefficient and path length showed that the BII had significantly higher clustering coefficient than comparable random networks (Fig. 3C) while having a comparable path length (2.34 compared to 2.14 for random networks). This finding is consistent with a small-world organization within the network that is verified by a high small-world coefficient (Fig. 3D). A small-world organization indicates the presence of a highway system of interactions that the majority of nodes use to interact with one another.

**Figure 4:**
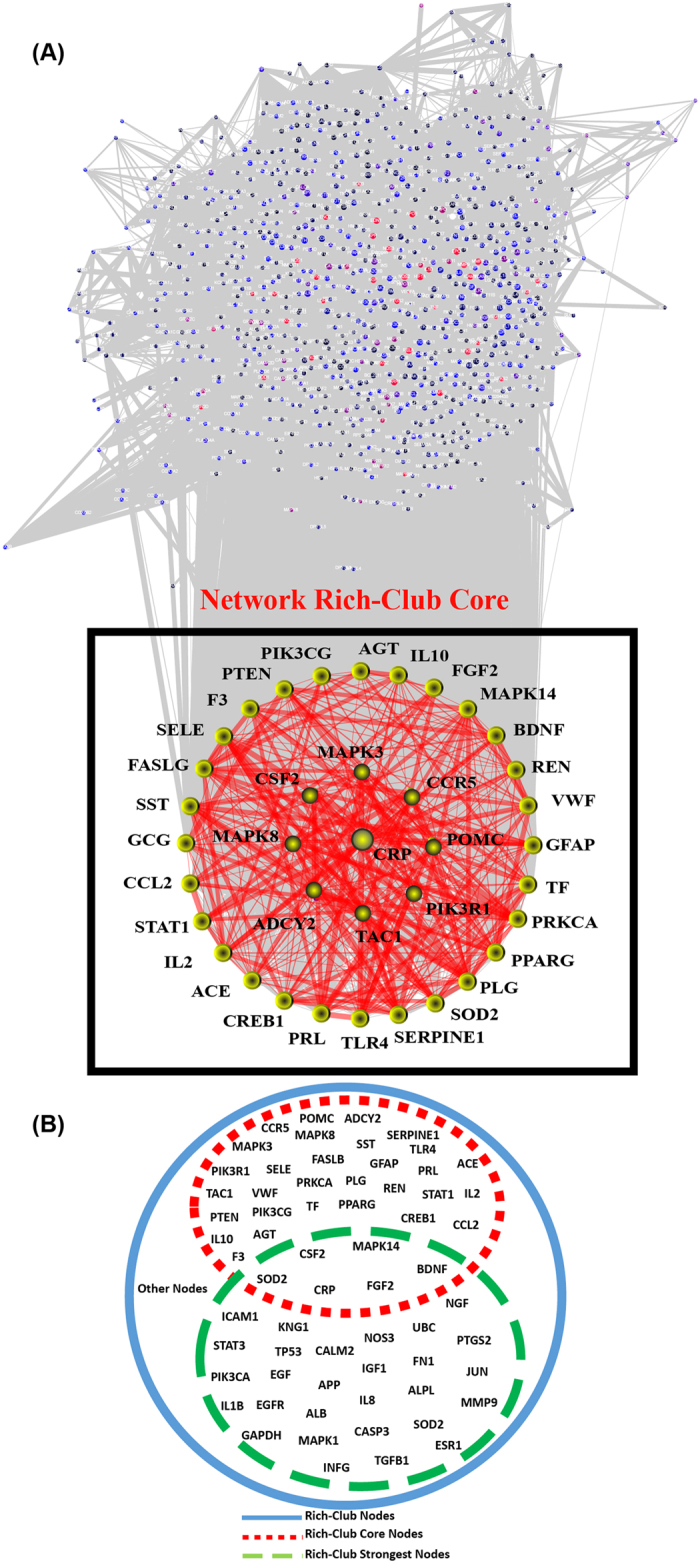
The core of BII network rich-club. (**A**) Network of brain ischemic interactome (BII) revealing the core of the rich-club (red box) and CRP as the center of the rich club. Only the core of the rich-club (subnetwork of nodes with degrees corresponding to the peak of normalized rich-club coefficient) is highlighted for illustrative purposes. Circles denote the protein nodes. Red edges label interactions are among rich-club proteins while grey edges label other interactions. Width of the edge maps the combined score of evidence for each interaction as per STRING database. The core of the rich-club shown in the square shows the dense interactions among the rich-club proteins. (**B**) Distribution of the BII nodes among the rich-club core and the strongest rich-club component.

We then assessed the presence of a rich-club organization within the BII. A rich-club organization in a complex network is characterized by nodes with high degrees that are heavily interconnected among each other compared to non-rich-club nodes. The presence of a rich-club within a network indicates that the rich-club nodes form a core sub-network that is most influential in the overall network. As shown in Fig. 3D, the BII network exhibits a rich-club organization characterized by increased rich-club coefficient (φ(k)) with increasing degree. The strongest component of the rich-club was identified where the φ(k) plateaus around 1. To investigate the significance of the discovered rich-club, we assessed whether this rich-club could be explained by the degree distribution of the network using a normalized rich-club coefficient (ρ(k)) comparing φ(k) to that of 1,000 randomly generated networks with similar degree distribution. The normalized rich-club coefficient (ρ(k)) reveals the presence of a significant rich-club between degrees 40 and 180 and peaking with a peak at degree 132. Interestingly, only one node, C-reactive protein (CRP) had the degree 132. The subnetwork of nodes with degrees corresponding to the highest normalized rich-club coefficient (above 1.3) are highlighted in Fig. 4A and defined as the rich-club core. Comparison of rich-club core nodes to that of the strongest rich-club component of the network revealed the presence of six overlapping nodes shown in Fig. 4B.

Comparison of rich-club components to non-rich-club components for frequency of encounter in the curated literature showed that the frequency of rich-club nodes was four-fold higher (Fig. 3C). In addition, members of the rich-club were found to span multiple pathophysiological pathways that predominantly included inflammatory and immunological response mechanisms summarized in Table 1.

### Functional and Topological Modules in Brain Ischemia Interactome

**Figure 5:**
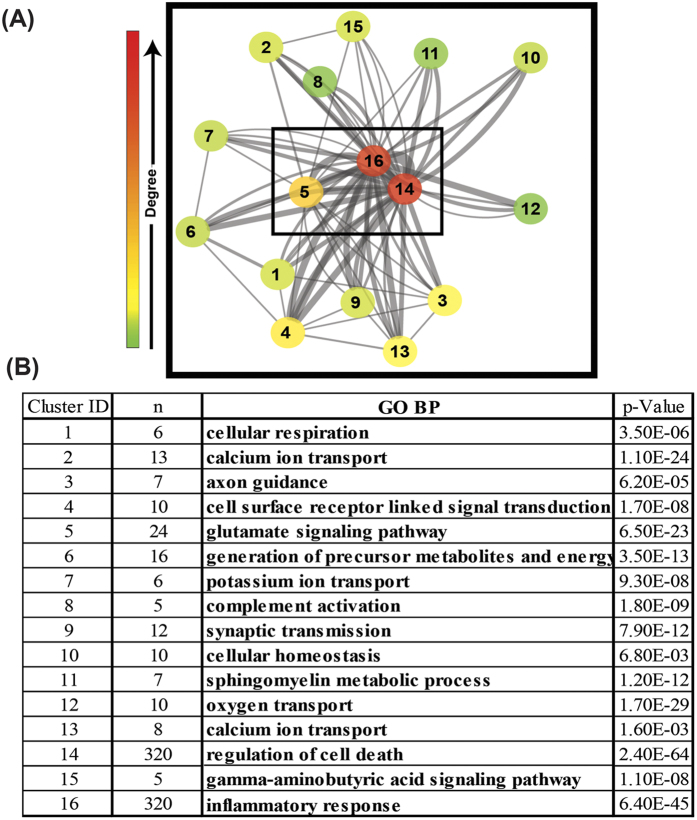
Identification of modules within the BII using Markov Clustering Algorithm. (**A**) Visualization of the network of interactions among the 16 MCL modules reveals that modules 5, 14 and 16 are the most central modules. Node color reflects the degree centrality measure and edge width denotes the number of connections among members of respective modules. (**B**) Functional annotation of GO biological processes predominantly enriched in each pathway showing multiple pathways interacting together in the context of brain ischemia/reperfusion injury (n: number of nodes in each cluster, p-value for the significance of enrichment of respective GO biological process. Clustering shown in (3A&3B) provides a faithful abstraction of the large network of protein interactions and emphasizes minor contributors that are otherwise masked in the full network analysis.

A full visualization of the curated BII involves massive interaction data that is not amenable to humane analysis (shown in Fig. 4A). Clustering the interactome to show interactions among clusters of proteins allows for simpler visualization and analysis of the interactions among prominent topological modules within the BII. The use of Markov Clustering Algorithm (MCL) identified 16 distinct modules with size five or more nodes within the BII. Figure 5A shows a reduced form of the interactome abstracted as interactions between major MCL modules. Functional annotation of enriched GO biological processes in each cluster is summarized in Fig. 5B and shows that these topological modules are also functionally distinct, each enriched for a specific pathophysiological pathway. Analysis of the degree of each module showed that modules 5, 14 and 16 are the most central modules. These modules are enriched for inflammatory response, regulation of cell death and glutamate receptor signaling and serve as a core highway that interconnects multiple pathological and homeostatic cell responses that occur after cerebral ischemia.

### Estrogen: a Pleiotropic Effect in Stroke Treatment

**Figure 6:**
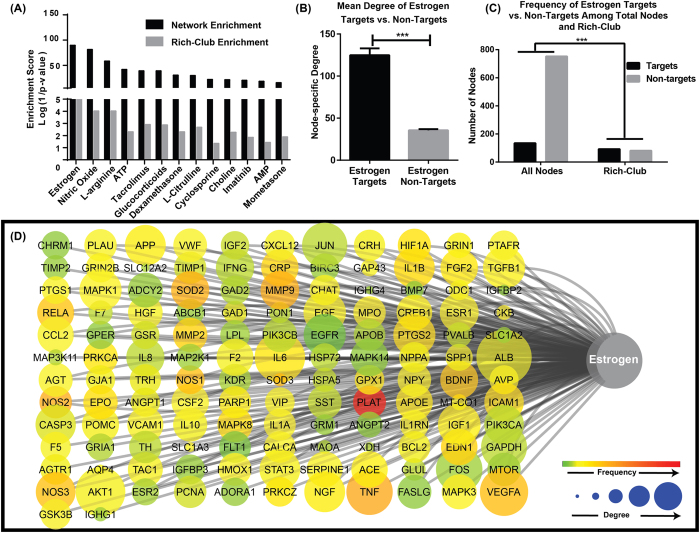
Estrogen targets within the BII showing that estrogen preferentially targets components of the rich-club. (**A**) Enrichment scores for the different drugs and chemicals that target the network and the rich-club. Black bars show enrichment scores for targets in the network. Grey bars show enrichment scores for targets in the rich-club. (**B**) Mean degree of estrogen targets is significantly higher than estrogen non-targets (*Bars* = *mean* +/− SEM. *p < *0.0001*). (**C**) Distribution of estrogen targets and non-targets within the entire BII network and the rich-club revealing a preference of estrogen to target rich-club components. Enrichment of estrogen targets in the rich-club was assessed by Fischer exact t-test *p < *0.0001. Bars* = *mean* +/− *SEM.* (**D**) Different targets of estrogen among the BII. Nodes other than estrogen are encoded by color (denoting frequency of occurrence in literature) and size (denoting degree in the BII network).

In the last step, we used the findings of the network analysis as a screening effect for potential therapeutics. Analysis of protein-drug interactions, performed through STITCH (Search Tool for Interactions of Chemicals)^16^ and GeneCodis (Gene Annotation Co-occurrence Discovery)^17^, revealed estrogen as the most enriched chemical therapeutic within our BII (Fig. 6A). Targets of estrogen within our BII are shown in Fig. 6D and include up to 15% of the nodes in the network. Estrogen was also found to preferentially target nodes within the rich-club (53% of total rich-club components) which was reflected by a significant enrichment on Fischer Exact t-test (Fig. 6C). Eventually, estrogen targets were found to have significantly higher degrees compared to estrogen non-targets (Fig. 5B). In addition estrogen was found to selectively target the three central pathological modules in the network (96% of estrogen targets) that include apoptosis, inflammatory response and glutamate excitotoxicity. Besides estrogen, other chemical compounds that have similar pleiotropic effect (beneficial or harmful) on targeting components of the BII are shown in Fig. 6A and include nitric oxide (and its donor l-arginine), ATP, tacrolimus, glucocorticoids and others.

## Discussion

The main findings of this study are the detection of a rich-club organization within the brain ischemia protein interaction network and the use of network analysis to identify prominent interacting pathways in disease pathophysiology. As a topological measure, rich-club organization of a network occurs when nodes with high degrees are heavily interconnected compared to nodes with lower degrees. A rich-club organization in the context of a disease-related protein interaction network indicates the presence of a pathological powerhouse that includes the most influential components on the structure of the system. Those rich-club components would serve as the primary targets for therapeutic intervention or as putative prognostic biomarkers. C-reactive protein (CRP), cytokines and chemokines (CCR5, IL10, IL1B, IL2), growth factors (BDNF, FGF2) cell signaling molecules and transcription factors (e.g. STAT1, MAPK3/8/14, PPARG, PIK3CG) were among the central hubs in the network that formed the rich-club (Fig. 4). Our findings that the rich-club covers multiple pathogenic pathways confirm previous literature that a multitude of pathophysiological mechanisms come into play after ischemic stroke and determine the functional outcome^4^,^5^,^18^,^19^. We further demonstrate using network analysis of the BII that those different pathogenic mechanisms communicate using a core of hub proteins to shape the overall outcome in ischemic stroke (Table 1). Although the identified rich-club is a novel finding of this paper, the fact that rich-club proteins were also more frequently reported in the literature indicates that research was independently centered on this rich-club prior to our discovery. Our findings are independent from literature since the frequency of occurrence for each protein was not included in network analysis to avoid literature bias in our study.

Few reports had previously investigated the presence of rich-club organization in protein interaction networks^9^,^20^,^21^,^22^,^23^. For instance, McAuley *et al.* studied the protein interaction network of *Saccharomyces cerevisiae* and found that there is no rich-club organization. Their finding indicates that proteins in the system of the studied yeast have modular function not centered on a high-capacity hub center. However, previously studied protein interaction networks still exhibit a similar power-law degree distribution and small-world organization as seen in the BII network^24^,^25^. In a different setting, a rich-club organization was recently discovered in the network of the brain connectome by the work of Sporns and colleagues and is thought to provide a better understanding of complex neuronal connectivity in the brain using data from multiple imaging techniques. The discovered rich-club organization in the brain connectome was then studied for variability during development and disease^8^,^26^.

Among the multiple interacting pathways after cerebral ischemia, the complement and coagulation cascade (CCC) was the most enriched pathway in the BII. Complement and coagulation proteins are two proteolytic cascades of the innate immune system and can cross-activate one another^27^,^28^. Both pathways are central to both ischemia (endothelial activation and formation of clot) and reperfusion (dissolution of clot and binding of complement components) and have been previously shown to be amongst the first players after ischemic stroke^3^,^15^,^18^. In addition, CRP, a component of the CCC pathways was among the most significant core of the rich-club. The fact that the center of the rich-club, CRP, as well as other components are recognition molecules and acute-phase reactants indicates that the rich-club is activated first and then it stimulates a diverse network of other interacting partners leading to the overall stroke pathogenic network. This finding supports the previously reported early role of the CCC pathway in initiating and exacerbating injury after stroke onset, and is in line with the current evidence on the role of CRP and the CCC pathway in stroke pathogenesis^3^,^15^,^18^. Previous reports have shown that CRP binds exposed phosphocline on damaged or stressed cells and mediate activation of C1q, the initiator component of the classical complement pathway^29^. Similarly, other components of the complement pathway serve at the recognition front for cell stress and injury secondary to activation by newly expressed surface antigens on ischemic cells and binding or by binding of natural IgM antibodies^15^. Prior to this study, the complement system was hypothesized to serve a hub-like position in inflammatory and homeostatic mechanisms^30^. Eventually, our findings have quantitatively confirmed the role of the CCC pathway in early recognition of injury and activation of consequent pathways. In addition, the central and early role of CRP in ischemic stroke pathogenesis may explain why CRP serves as an early independent prognostic marker of recovery and mortality after ischemic stroke^31^,^32^,^33^,^34^,^35^,^36^, and why CRP injection increases cerebral infarct in experimental stroke^37^. Moreover, the coagulation pathway, the other branch of the CCC, is also a major contributor to early response to infract through proteolytic activation of the complement system and other pathways as well as through thrombolysis and micro-emboli formation.

Our analysis of the modular organization of the entire BII revealed that despite the central role of the CCC pathway, other prominent pathophysiological pathways are the major integrative core of the pathophysiological processes including apoptosis, inflammation, glutamate excitotoxicity. These three pathways were enriched in the three most central modules in the network and were heavily interconnected amongst one another and with other modules in the network. This finding provides supporting evidence for the current stroke pathogenesis model that includes inflammation, cell death and excitotoxicity as the three hallmarks of brain ischemia^18^. In addition to the core modules in the BII, the presence of diverse pathophysiological processes indicates that regardless of how secondary injury after stroke is initiated, the spectrum of pathophysiological processes involved are more complex and diverse requiring a multi-target intervention that can reduce the pathogenic activity of the rich-club powerhouse. However, such multi-target intervention would specifically benefit from the analysis of modules within the network through preferentially targeting modules that contribute to pathological effects (such as Glutamate excitotoxicity, apoptosis, and inflammation) versus homeostatic and reparatory modules (such as axon guidance, cellular respiration and cellular homeostasis) (Fig. 5B).

Through the integration of data from drug-protein interaction databases, estrogen was detected as intervention with most enriched targets within the network compared to other drugs screened in this study. Estrogen targets were also preferentially enriched within the rich-club and specifically targets the central pathological modules of BII, a finding that comes in accordance with accumulating preclinical evidence on the neuro-protective role of estrogen^38^,^39^,^40^,^41^. Through both its genomic and non-genomic effects, estrogen is believed to be the reason behind the sex differences in vulnerability and outcome of stroke as women are more protected prior to menopause^40^,^42^,^43^.

Despite that, estrogen has failed to provide any therapeutic benefit in trials on post-menopausal (PMN) women^44^. However, this effect should not challenge the predicted neuroprotective effects of estrogen by preclinical work and trials on perimenopausal women^45^. In fact, trials on PMN estrogen replacement in the context of stroke suffers from mistranslation^38^,^41^. Given the data presented in this report, we recommend that estrogen treatment should be better exploited in the field of stroke and suggest that the exploitation of the curated network will help better explain the molecular effects of estrogen and the potential strategies that enhance its efficacy. Adverse outcomes after estrogen treatment relate to the inappropriate time, dose and target population of treatment. In fact, the impact of estrogen on stroke recovery should be assessed in the correct system-level window of estrogen efficacy, i.e. prior to the aging of estrogen response factors. The loss of estrogen efficacy in PMN women is a phenomenon possibly secondary to the aging and changes of estrogen responsive signaling molecules that occurs after menopause. One example of such elements is Growth Hormone/Somatomedin C (IGF-1) axis that exhibits significant age-related changes in PMN women often referred to as “somatopause”^41^. Not surprisingly, our analysis showed that IGF-1 is a prominent component of the rich-club and a high degree target for estrogen in BII. A potential future comparison of PMN-changes related interactome with the current BII may reveal significant pathophysiological mechanisms behind estrogen’s deleterious effects in the post-menopausal period.

Although we have focused our discussion on estrogen, the most enriched drug in the network, other drugs were found to exhibit pleotropic influence on the rich-club of BII, yet such pleiotropic effect may not necessarily mean a perfect therapeutic intervention since influencing the rich-club may affect both reparatory and pathogenic mechanisms. Interestingly, drugs with targets enriched in our rich-club are currently tested for efficacy in stroke trials which emphasizes the utility of our tool to extract valid information from mass literature. One other example from our analysis is nitric oxide (NO) and its donor l-arginine whose targets were significantly enriched within the rich-club; however, till now there is no clear evidence on the therapeutic effect of NO donors on stroke. A possible answer to this may come from the recently completed Efficacy of Nitric Oxide in Stroke (ENOS) Trial^46^.

Grouped with the previous literature on stroke pathophysiology^3^,^15^,^18^, the findings suggest that acute stroke therapy may benefit from CCC pathway interruption through thrombolysis and inhibition of damage-associated signaling molecules of the immune system. This finding is in line with the current standard of care for acute stroke patients involving a rapid infusion of thrombolytic t-PA; yet, further investigation of efficacy and safety of immune-modulatory interventions in preventing exacerbation of early injury is still required. The properties of the rich-club in the BII have also revealed that diverse pathological pathways might come into play after the onset of ischemia and that drugs with pleiotropic effects are recommended to be considered. Here, we report several pleiotropic compounds that act on the rich-club and are candidates for potential consideration in stroke therapy while focusing on estrogen, the drug with most enriched targets in the network.

This study only included therapeutic candidates provided in the GeneCodis and STITCH databases. Future work will utilize the data provided in this network to assess the effect of other therapeutics as well as that of combination of interventions on the overall network properties. An ultimate aim will be to provide a tool for investigators to provide *in-silico* to probe the effects of potential and novel therapeutics on the overall disease pathophysiology as well as on specific pathways in disease pathogenesis. Another limitation of this study is the absence of data on the direction and type of change in protein levels and regulation as well as the timing of this change. Curation of this data is part of an ongoing effort to help perform more detailed predictions of the effects of different interventions and allow for *in-silico* replication of experimental scenarios relevant to stroke pathogenesis and therapy. In addition, curation of other disease interactomes will also allow cross-disease comparative analysis to provide candidate disease-specific biomarkers and understand common mechanisms of pathogenesis.

In conclusion, the approach used in this paper to curate preclinical and clinical data to better understand complex diseases and form an *in-silico* screening tool for therapeutics is a novel introduction to bioinformatics research and may have future applications in a variety of other diseases.

## Methods

### Extraction of Target Dataset

Literature on brain ischemia was extracted from PubMed using the MeSH term (Brain Ischemia) and the search key (Stroke OR brain infarct* OR cerebral infarct* OR brain ischem* OR cerebral ischem* OR ischemic brain injury) as well as from references of reviews and extracted papers. Abstracts were first screened for relevance by two investigators using the title. Selected abstracts were then processed into an annotator tool (designed by authors) for extraction of protein and gene terms reported in association with ischemic stroke. Supplementary Figure 1 illustrates the selection process in details.

### Text Annotation and Accession Mapping Tools

To extract protein and gene names and identifiers from the text, we adopted a semi-automatic annotation protocol using an in-house annotator to ensure specificity and sensitivity of capture. The tool (1) extracts different versions of gene and protein names from UniProt (Universal Protein Resource) and HGNC (HUGO Gene Nomenclature Committee) databases, (2) checks the text of the extracted abstracts with the gene and protein terms extracted from the databases to annotate exact matches, and (3) detects and annotates close matches based on variations of the extracted terms defined by the authors in the form of computational rules (Supplementary Figure 2). The detected and annotated terms were extracted into separate datasets, each identified by the ID of the source paper. A human annotator with experience in both stroke and proteomics verified the captured terms and inspected the abstracts using the same annotator tool for additional terms. The original database was updated with new terms introduced by the human annotator. Then, an in-house C# program communicated with the UniProt and HGNC to retrieve human orthologs of captured terms and their corresponding accessions. The frequency of each accession was calculated as the number of distinct reports mentioning the accession in question. Supplementary Figure 2 illustrates the details of the text annotation process leading to the captured set of accessions.

### Functional Annotation and Interactome Data

The list of accessions retrieved from literature was functionally annotated using DAVID^13^ for GO cellular component, GO biological processes, KEGG pathways, tissue expression and the Genetic Association of Diseases database. DAVID is a tool that allows for identification of over-represented terms and categories in a subset of genes or proteins and identifies enrichment scores for annotations. Protein-protein interactions (PPI) were obtained from STRING database that includes data on interacting proteins or genes within each species. Interactions among the list of curated accessions were retrieved using a threshold score of 0.4^11^. The resulting network included 17,425 binding interactions among 886 proteins.

PPI data obtained from STRING was then mapped into the full Brain Ischemia interactome using Cytoscape 3.1.1^11^,^47^, a graph and network analysis and visualization tool. In addition, data on protein-drug interaction was retrieved from GeneCodis and STITCH. GeneCodis provides enrichment analysis of gene-drug interactions within a network using PharmGKB database^17^ while STITCH allows for analysis of chemical-gene interactions within a network using a dataset of 3 million chemical agents^16^. Drug or chemical targets were defined in our study as those proteins and genes with which the drug or chemical interacts and affects regulation. Supplementary Table 2 describes the different tools and resources used in the functional annotation process.

### Interactome Graph Analysis

#### Graph Measures

Examination of the topology of the BII network using graph theory was performed through the Systems Biology and Evolution MATLAB Toolbox (SBEToolbox) and Cytoscape^47^,^48^. Characteristic measures of network organization were computed including node-specific degree k, clustering coefficient, path length, betweenness centrality, and modularity. The power-law degree distributions and adjacency matrices of the networks were generated using MATLAB (Mathworks, R2013a).

#### Rich-Club Analysis

The emphasis of this work is the detection of a rich-club organization among the nodes within the network of the BII. A rich club is a set of high-degree nodes that are more densely interconnected than predicted by the node degrees alone^20^. A rich-club coefficient φ(*k*) is computed over the range of degrees in the network as previously described by Colizza *et al.*^20^. For a given degree distribution {*k*_1_, *k*_2_, …, *k*_n_}, rich-club coefficient for each degree *k* is calculated as the number of edges among nodes with degrees higher than *k* divided by the maximum possible number of edges among those nodes:

φ(*k*) = 2*E_>*k*_/((N_>*k*_)*(N_>*k*_ − 1)), where N_>*k*_ is the number of nodes with a degree higher than *k*, and E_>*k*_ is the number of edges among those nodes.

To calculate normalized rich-club coefficient, we generated 10,000 random networks with the same degree distribution as the network of interest as described by Viger and Latapy^49^. The average of rich-club coefficients of the random networks φ_random_(*k*) is calculated, and the normalized rich club is computed as:


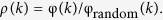


The normalized rich-club coefficient was calculated from the lowest degree to the second highest degree encountered in the BII. When the normalized rich-club coefficient ρ(k) is greater than 1, it indicates the rich-club organization in the network is significant and cannot be explained by the degree distribution of the network alone^20^,^50^.

#### Clustering

Markov Clustering of BII network was performed through MATLAB SBEToolbox^48^ following the previously described Markov clustering (MCL) algorithm^51^. Prominent modules were visualized through Cytoscape. Functional annotation of enriched GO biological processes within each module was performed in DAVID.

### Statistics

Statistical analyses were performed through GraphPad Prism 6 (GraphPad Software, Inc.). Numerical data and histograms were expressed as the mean ± S.D. Two-tailed Student’s t-test was used to compare the difference in frequency of rich-club vs. non-rich –club nodes as well as to compare the rich-club coefficients of estrogen targets vs. non targets. Fischer exact t-test was used to calculate p-value for enrichment of annotation terms and drug targets between the network and the rich-club.

## Additional Information

**How to cite this article**: Alawieh, A. *et al.* A Rich-Club Organization in Brain Ischemia Protein Interaction Network. *Sci. Rep.* **5**, 13513; doi: 10.1038/srep13513 (2015).

## Supplementary Material

Supplementary Information

## Figures and Tables

**Table 1:** KEGG pathways significantly enriched in the rich-club sub-network.

KEGG Pathway	Gene Count	P-Value
Chemokine signaling pathway	42	3.2E-19
Cytokine-cytokine receptor interaction	46	1.1E-16
Toll-like receptor signaling pathway	29	3.7E-16
Apoptosis	26	6.8E-15
Focal adhesion	37	6.1E-14
ErbB signaling pathway	24	6.3E-13
Neurotrophin signaling pathway	28	9.0E-13
GnRH signaling pathway	25	1.2E-12
T cell receptor signaling pathway	26	1.5E-12
Fc epsilon RI signaling pathway	22	4.6E-12
NOD-like receptor signaling pathway	19	4.1E-11
VEGF signaling pathway	20	1.6E-10
Jak-STAT signaling pathway	28	2.2E-10
Calcium signaling pathway	29	8.9E-10
Leukocyte transendothelial migration	23	3.1E-09
MAPK signaling pathway	35	5.3E-09
Adipocytokine signaling pathway	17	1.2E-08
Gap junction	19	2.4E-08
Phosphatidylinositol signaling system	17	5.5E-08
Aldosterone-regulated sodium reabsorption	13	7.5E-08
Progesterone-mediated oocyte maturation	18	8.6E-08
Complement and coagulation cascades	16	1.4E-07
Melanogenesis	19	1.4E-07
mTOR signaling pathway	14	1.7E-07
Natural killer cell mediated cytotoxicity	21	6.9E-07
Long-term depression	14	5.3E-06
Fc gamma R-mediated phagocytosis	16	9.8E-06
Long-term potentiation	13	2.5E-05
RIG-I-like receptor signaling pathway	13	3.9E-05
Insulin signaling pathway	17	1.8E-04

Modified Fisher Exact P-Value used here was calculated through DAVID annotation tool. Pathways with a p-value less than 0.001 are displayed.
