# Supplementary material for: A Rich-Club Organization in Brain Ischemia Protein Interaction Network
Source: Sci Rep. 2015 Aug 27;5:13513. doi: 10.1038/srep13513 (PMC4550934; doi:10.1038/srep13513)
Supplement: Supplementary Information [file srep13513-s1.pdf]

Supplementary Material

A Rich-Club Organization in Brain Ischemia Protein Interaction Network

Ali Alawieh [1,2] , Zahraa Sabra [1,2], Mohammed Sabra [2], Stephen Tomlinson [3], and Fadi Zaraket [2] *

[1]Department of Neurosciences, Medical University of South Carolina, Charleston, SC 29425; [2]Department of Electrical and Computer Engineering, American University of Beirut, Beirut, Lebanon; [3]Department of Microbiology and Immunology, Medical University of South Carolina, Charleston, SC 29425

***Correspondence to:** Fadi A Zaraket, PhD; Dept. Electrical & Computer Engineering, American University of Beirut, Beirut, Lebanon. Tel: +961(1)350-000 ext 3484. Fax: +961(1)744-462. Email: fz11@aub.edu.lb

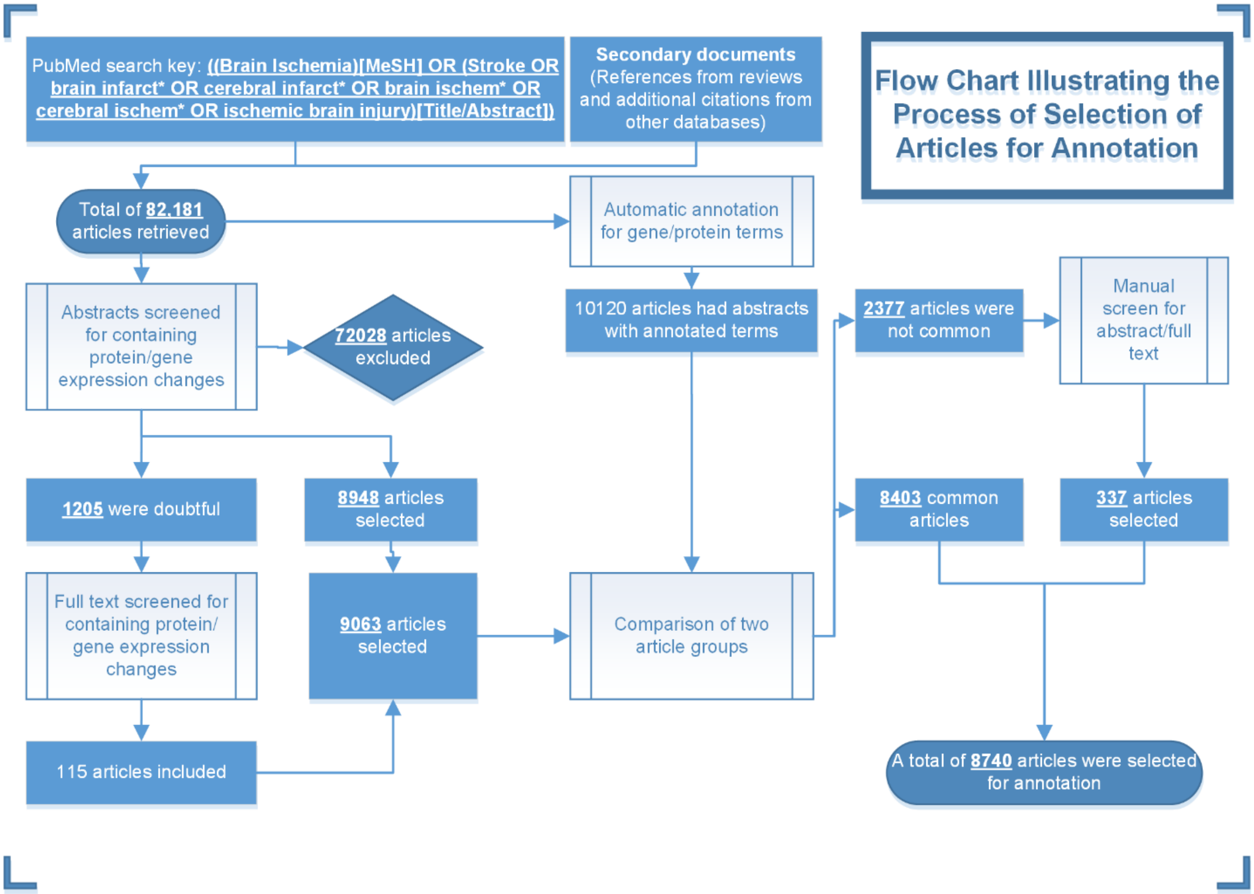

Supplementary Figure 1

Supplementary Figure 1 - Flow Chart Illustrating Article Selection Process. Starting from around 82,000 articles retrieved from literature search, 8740 articles are included in the annotation process after both manual and automatic selection. The main inclusion criteria for a given article is the presence of information on gene or protein expression changes in the abstract. Articles selected at this step is passed to our annotator tool for term annotation and mapping as described in Supplementary Figure 2.

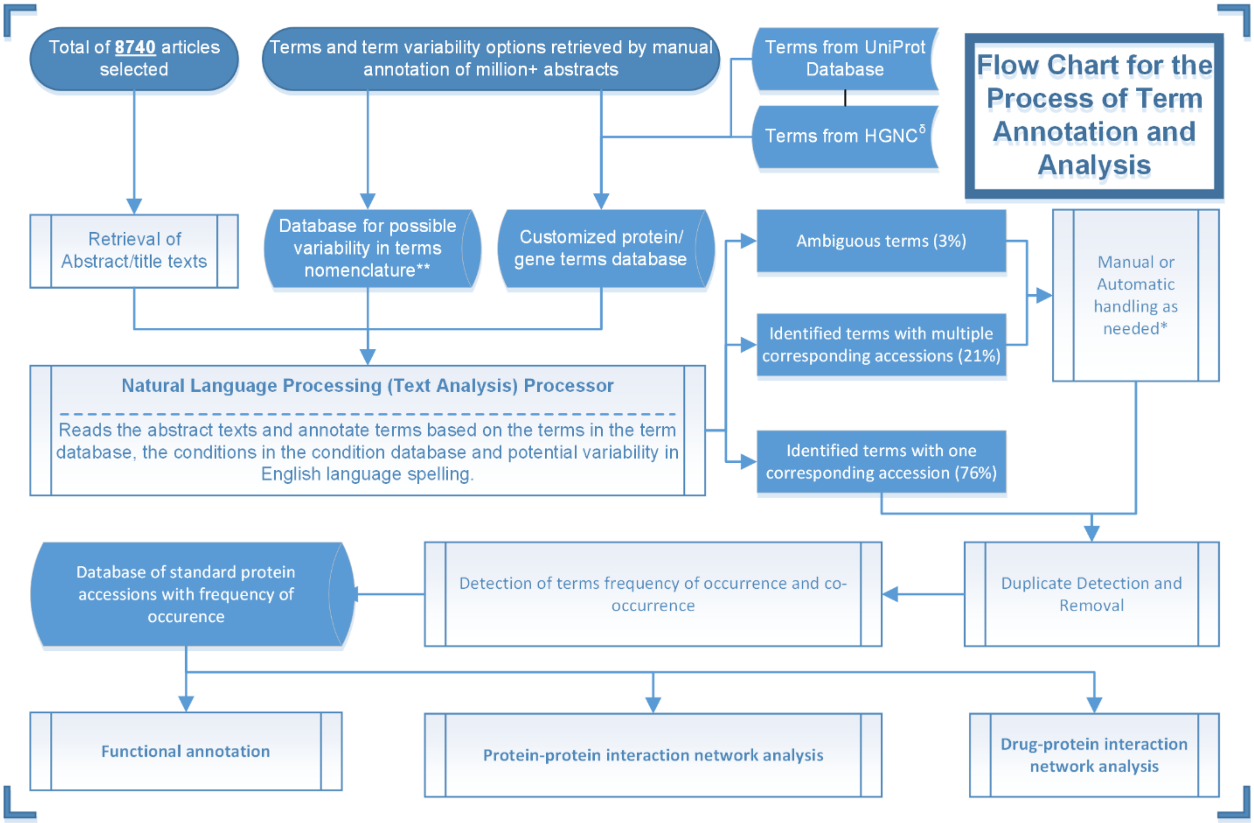

Supplementary Figure 2

Supplementary Figure 2 - Flow Chart Illustrating Protein Annotation Process. $^{\delta}$ HGNC: HUGO Gene Nomenclature Committee. *: Manual or automatic handling involves selection of which of the ambiguous terms is truly a protein as well as which accessions are the ones referred to by a given term with multiple relevant accessions. The first option is treated using manual examination of the terms and assignment of relevant accessions if any. The latter option is managed automatically through detecting other terms in the abstract that may accurately describe the term of interest and un-resolved cases are then manually handled. Other steps not mentioned in the figure include network visualization through Cytoscape, and the scripts used to convert accessions from one form to another and communicate with different annotation databases. **: Possible variability in term include among others: singular vs. plural, multiple numbers or characters after the name indicating subunits, minor spelling errors like switching order of vowels, and presence or absence of slash/hyphen/brackets. Abbreviations in the absence of full terms were handled depending on context. In addition, proteins with lower frequency of occurrence in literature (less than 5 unique reports) are manually verified.

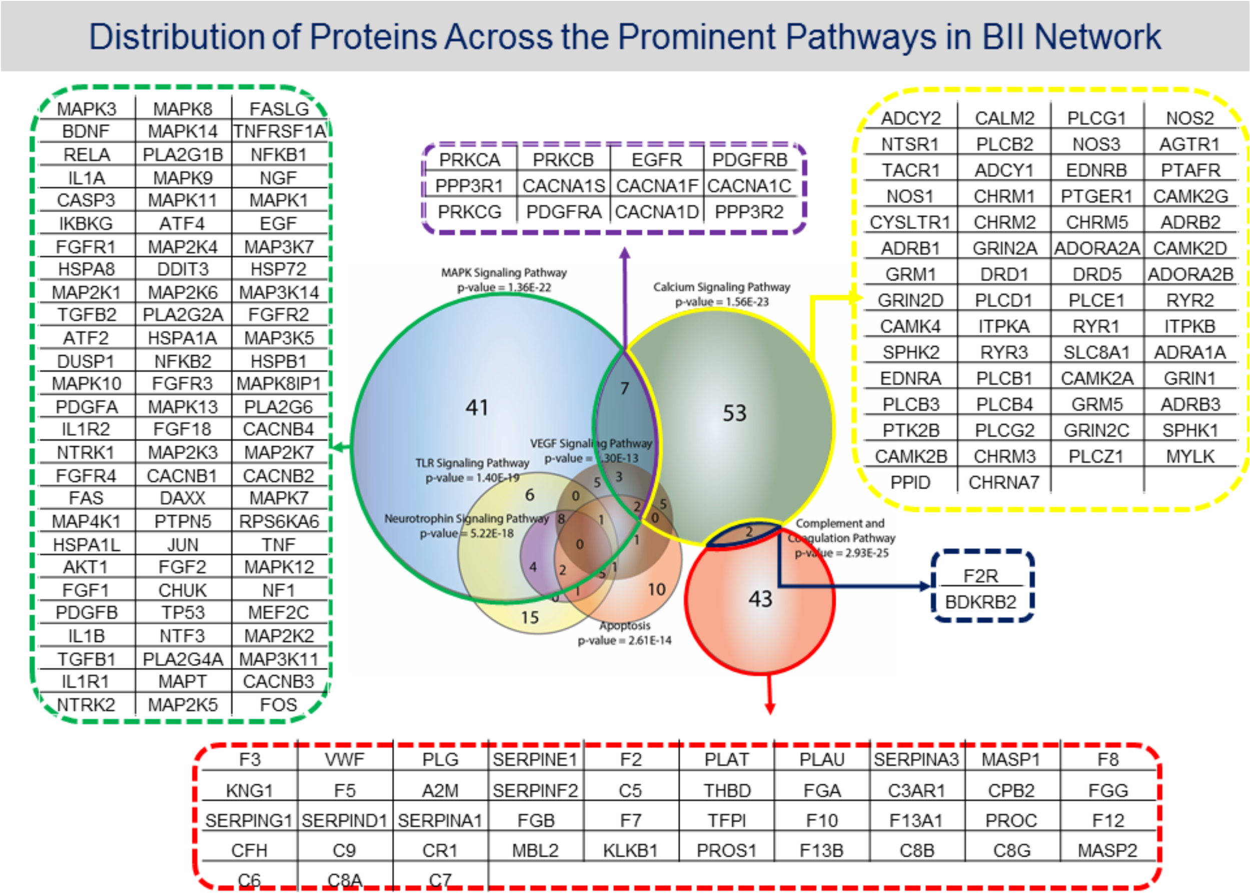

Supplementary Figure 3

Supplementary Figure 3 – Distribution of proteins across the different prominent pathways in BII network.

Supplementary Table I. Description of tools and databases used in our analysis.			
**Database/ Tool**	**Full Name**	**Description**	**Link**
UniProt	The Universal Protein Resource	Comprehensive resource of protein sequence and annotation data.	http://www.uniprot.org/
HGNC	HUGO Gene Nomenclature Committee	Resource for gene nomenclature, gene families and associated resources including links to genomic, proteomic and phenotypic information	http://www.genenames.org/
DAVID	The Database for Annotation, Visualization and Integrated Discovery	Web application that provides a comprehensive set of functional annotation tools for proteomic and genomic analyses	http://david.abcc.ncifcrf.gov/
GO	Gene Ontology	A vocabulary database that describes gene products in terms of their associated biological processes, cellular components and molecular functions in a species-independent manner.	http://geneontology.org/
KEGG Pathway	Kyoto Encyclopedia of Genes and Genomes	A collection of manually drawn pathway maps representing our knowledge on the molecular interaction and reaction networks	www.genome.jp/kegg/
STRING	Search Tool for the Retrieval of Interacting Genes/Proteins	STRING is a database of known and predicted protein interactions. The interactions include direct (physical) and indirect (functional) associations	http://string-db.org/
GeneCodis	Gene Annotation and Co-occurrence Discovery	GeneCodis is a web-based tool for the ontological analysis of large lists of genes.	http://genecodis.cnb.csic.es/
PharmGKB	The Pharmacogenomics Knowledge Base	The Pharmacogenomics Knowledge Base, PharmGKB, is an interactive tool for researchers investigating how genetic variation effects drug response.	https://www.pharmgkb.org/
STITCH	Search Tool for Interactions of Chemicals	STITCH is a resource to explore known and predicted interactions of chemicals and proteins. Chemicals are linked to other chemicals and proteins by evidence derived from experiments, databases and the literature.	http://stitch.embl.de/
Cytoscape	Cytoscape	A software platform for visualizing complex networks and integration of annotation data	http://www.cytoscape.org/
SBEToolbox	MATLAB Systems Biology & Evolution Toolbox	A toolbox developed in MATLAB that employs algorithms to create random networks and to deduce clusters in the network	http://www.bioinformatics.org/sbetoolbox/

Supplementary Table I: Description of tools and databases used in our analysis.

Accession	Name	Name	Freq	Associated KEGG Pathways
P00750	**Plasminogen activator, tissue**	PLAT	879	Complement and coagulation cascades
P01375	**Tumor necrosis factor**	TNF	302	Cytokine-cytokine receptor interaction, mTOR signaling pathway, Focal adhesion, NF-kappa B signaling pathway, MAPK signaling pathwayApoptosis
P35228	**Nitric oxide synthase 2, inducible**	NOS2	297	Calcium signaling pathway, HIF-1 signaling pathway
P00441	**Superoxide dismutase 1, soluble**	SOD1	285	Peroxisome, Amyotrophic lateral sclerosis,<br>Huntington's disease
P14780	**Matrix metallopeptidase 9**	MMP9	276	TNF signaling pathway, Leukocyte transendothelial migration, Estrogen signaling pathway
P14136	**Glial fibrillary acidic protein**	GFAP	268	--
P15692	**Vascular endothelial growth factor**	VEGF	246	
O43915	**C-fos induced growth factor**	FIGF	242	Cytokine-cytokine receptor interaction, mTOR signaling pathway, Focal adhesion
P29475	**Nitric oxide synthase 1**	NOS1	240	Calcium signaling pathway, Phagosome, Metabolic pathways, Long-term depression
P19838	**Nuclear factor of kappa light polypeptide gene enhancer in B-cells**	NFKB	227	MAPK signaling pathway, NF-kappa B signaling pathway, Apoptosis,<br>Toll-like receptor signaling pathway, Chemokine signaling pathway,<br>HIF-1 signaling pathway
Q04206	**V-rel avian reticuloendotheliosis viral oncogene homolog A**	RELA	226	MAPK signaling pathway, NF-kappa B signaling pathway, Apoptosis,<br>Toll-like receptor signaling pathway, Chemokine signaling pathway,<br>HIF-1 signaling pathway
P23560	**Brain-derived neurotrophic factor**	BDNF	193	MAPK signaling pathway, Neurotrophin signaling pathway

P08253	**Matrix metallopeptidase 2**	MMP2	181	Leukocyte transendothelial migration, GnRH signaling pathway, Estrogen signaling pathway
P05305	**Endothelin 1**	EDN1	179	HIF-1 signaling pathway, TNF signaling pathway
P02741	**C-reactive protein, pentraxin-related**	CRP	173	--
P01584	**Interleukin 1, beta**	IL1B	158	MAPK signaling pathway, Cytokine-cytokine receptor interaction, Apoptosis, Toll-like receptor signaling pathway, NOD-like receptor signaling pathway, Cytosolic DNA-sensing pathway
P45983	**Mitogen-activated protein kinase 8**	MAPK8	146	MAPK signaling pathway
P45984	**Mitogen-activated protein kinase 9**	MAPK9	143	MAPK signaling pathway

Supplementary Table II: Summary of the most common proteins studied in stroke literature. Frequency is defined as the number of unique original reports (excluding reviews) reporting the corresponding protein.
